# Radiation therapy induced intestinal barrier damage and repair process - differences in salivary metabolites and monitoring of intestinal barrier function

**DOI:** 10.3389/fimmu.2025.1590219

**Published:** 2025-06-12

**Authors:** Zhang Jingjing, Wang Kun, Qiao Yanyu, Zhang Mengjie, Chen Yunqing, Tian Yulong, Jiao Xuelong, Tan Xiaojie, Jiang Haitao, Hou Feng

**Affiliations:** ^1^ Department of Pathology Department, Affiliated Hospital of Qingdao University, Qingdao, China; ^2^ Department of Gastrointestinal Surgery, Affiliated Hospital of Qingdao University, Qingdao, China; ^3^ Department of Gynaecology, Affiliated Hospital of Qingdao University, Qingdao, China

**Keywords:** rectal cancer, radiotherapy, damage and repair, intestinal mucosal barrier, biological barrier, immune barrier, salivary metabolites

## Abstract

**Purpose:**

Colorectal cancer (CRC) is still one of the most common malignant tumors, with gradual increase in its annual morbidity and mortality. But most cases are diagnosed in the late stage. For stage II-III cancer, clinical guidelines recommend surgery following neoadjuvant radiation therapy at ≥6 weeks after the last radiotherapy is completed. However, radiotherapy may impair intestinal mucosal barrier function, especially the biological and immune barriers, accompanied by perioperative complications. This study was conducted to investigate the changes, repair patterns, and potential mechanisms in patients after radiotherapy.

**Methods:**

This study detected inflammatory factors in postoperative intestinal mucosal tissue and serum, as well as metabolites in saliva samples, and collected hematoxylin-eosin (HE)-stained pathological images in CRC patients who had received and did not receive radiotherapy.

**Results:**

The results showed that after radiotherapy, there were significantly impaired intestinal mucosal tissue structure; obviously elevated inflammatory factors in intestinal mucosal tissue and blood; as well as upregulation/downregulation of metabolites in saliva samples.

**Conclusion:**

In conclusion, findings in this study may provide potential reference for predicting the recovery of intestinal mucosa and selecting the optimal timing for surgery after radiotherapy. In addition, this study will benefit the understanding and reduction of perioperative complications caused by intestinal barrier damage.

## Introduction

Colorectal cancer (CRC), as one of the most common malignant tumors, presents a rising incidence rate in recent decades (1). In 2020, CRC ranked among the top ten newly diagnosed cancers worldwide, with 1,944,826 cases (10.0%) reported (2). In China, the morbidity and mortality of digestive malignancies are still increasing (3), significantly compromising public health and life expectancy, while also imposing substantial economic and social burdens on families and society (4). Patients with advanced gastrointestinal cancers may still experience poor prognosis, despite advancements in cancer screening. The early stages of gastric, colorectal, and liver cancers in China account for a relatively low percentage of total cases, with most being diagnosed at more progressive stages (5). For patients with advanced CRC, neoadjuvant therapy radiotherapy may offer oncological benefits (6). Radiation therapy can have certain side effects on the intestinal tissue of patients, namely the occurrence of radiation-induced enteritis, which damages the intestinal mucosal barrier function of patients. However, radiotherapy can damage the intestinal mucosal barrier, especially the biological and immune barriers, potentially leading to bacterial translocation, which is closely associated with perioperative complications such as infection and anastomotic fistulae (7, 8). According to existing Guidelines (9), surgery should be performed wait at ≥6 weeks following radiotherapy. In our previous research, damage to the intestinal barrier, caused by radiotherapy, might be recovered in some cases within 8 to 12 weeks; however, it was impossible to restore to the normal status around three months post-treatment. Our previous research found that radiation therapy can cause changes in the oral microbiota of patients with rectal cancer (10). Therefore, this study was performed to analyze the damage to the barrier function and related biological changes of intestinal mucosa caused by radiotherapy. This study aims to assess improvement and recovery of intestinal mucosal barrier function through preoperative colonoscopy biopsy specimens and saliva analysis, with an expectation to provide valuable insights for non-invasive monitoring to reduce perioperative complications resulting from radiotherapy, and to facilitate the recovery of intestinal barrier function in CRC patients.

## Materials and methods

### Materials and grouping situation

This study postoperatively collected intestinal tissue samples from patients who underwent radical surgery for CRC at our Center between 2023 and 2024. Patients who underwent surgery without prior radiotherapy and those who had surgery 60 to 90 days following radiotherapy were categorized into four distinct groups: Group 1 (no radiotherapy), Group 2 (surgery 60 ± 3 days post-radiotherapy), Group 3 (surgery 75 ± 3 days post-radiotherapy), and Group 4 (surgery 90 ± 3 days post-radiotherapy). Meanwhile, 24 saliva samples were collected from these CRC patients, comprising 13 patients who did not receive preoperative radiotherapy and 11 patients who underwent surgery following radiotherapy. Exclusion criteria: patients with medical history of inflammatory bowel disease and autoimmune diseases, and those with history of chemotherapy and targeted therapy; patients who had received immunosuppressive drugs, hormones, or antibiotics within the two weeks prior to surgery. Furthermore, hematoxylin-eosin (HE)-stained pathological images were taken from 50 postoperative pathological slides of patients who underwent radical surgery for CRC at our Center within the past year. There were no statistically significant differences in gender, age and other baseline data among groups.

### Observation of intestinal mucosal damage

All samples were selected from normal intestinal mucosal tissue located more than 5cm away from the tumor and fixed in a 4% paraformaldehyde solution for 48 hours. After being dehydrated with ethanol, soaked in xylene, embedded in paraffin, and sectioned, the pathological results of intestinal histology were observed under the light microscope after staining with HE and the repair of intestinal mucosal damage was summarized and analyzed.

### Western blotting was used to detect IFN - γ and TGF - β in intestinal mucosal tissue

Dilute the sample with the prepared cell lysis buffer to the same concentration, and take an equal amount of sample buffer from a test tube with a protein mass of 70ug for later use. Before loading the samples onto the gel, they were heat treated at 95-100°C, and then cooled on ice for 5 minutes. The electrophoresis conditions included 20 min of constant voltage at 80V for the stacking gel and 80 min at 100V for the separating gel. The gel was taken out and soaked in the transfer buffer for 15 min. Filter paper and PVDF(Polyvinylidene Fluoride) membrane were prepared and placed in the transfer buffer and deionized water, respectively. The gel was then sandwiched between the filter paper, PVDF membrane, and filter paper, and the electrodes were placed on the layer. After removing air bubbles at each layer, the upper electrode was placed on the sandwich material, and a constant current of 200 mA was applied for 1h. The PVDF membrane was blocked with a 5% skimmed milk blocking solution at room temperature for 1 hour, after which the solution was discarded without washing. The membrane was then incubated with an appropriate amount of primary antibodies (INF-γ, CST, USA; TGF-β, CST, USA) against β-actin (Immunoway, USA) at a dilution of 1:4000, along with the blocking solution, on a shaker at 4°C overnight. The membrane was washed four times with PBST (Phosphate Buffered Saline Tween 20), with each wash lasting 5 minutes. Subsequently, a secondary antibody (horseradish peroxidase-conjugated antibody, Jackson, USA) at a dilution of 1:5000 was added to the membrane and incubated on a shaker at room temperature for 1 to 2 hours. Subsequently, thoroughly wash the membrane with PBST for 5 minutes, repeating this process five times. After applying the developer to the PVDF membrane, incubate it at room temperature for 1 minute, using a developer volume EP calculated at 0.1 mL/cm². Next, wrap the film with plastic film while minimizing bubble formation during the procedure. The film should then be rapidly exposed to X-ray film in a darkroom and processed using an automatic film processor. Prior to the appearance of the optimal frequency band, it is essential to continuously adjust the exposure time.

### The ELISA method is used to detect the inflammatory factors IL-1 β and IL-1 β in the blood IL-6, IL-17, INF-γ

We collected data from 80 patients who underwent radical CRC surgery at our center within the past year (excluding patients with incomplete records). Group 1 comprised patients who underwent surgery within 8–12 weeks post-radiotherapy (n=57), while Group 2 consisted of patients who underwent surgery without prior radiotherapy (n=23). Take about 2ml of blood sample, centrifuge at 3000rpm for 15 minutes on a centrifuge, and transfer the supernatant obtained to a 2.5ml EP(Eppendorf) tube for further use. All operations are performed on ice. According to the detailed instructions of the kit (Jingmei, Jiangsu, China), ELISA was used to detect the levels of inflammatory factors IL-1 β, IL-6, IL-17, and INF - γ.

## Non-targeted Metabolomics for Detecting Changes in Salivary Metabolites

### Sample preparation and extraction

The sample stored at -80°C refrigerator was thawed on ice and vortexed for 10 s. A 150 μL extract solution (ACN (Acetonitrile): Methanol = 1:4, V/V) containing internal standard was added into 50 μL sample. Then the sample was vortex for 3 min and centrifuged at 12000 rpm for 10 min (4°C). A 150 μL aliquots of the supernatant was collected and placed in -20°C for 30 min, and then centrifuged at 12000 rpm for 3 min (4°C). 120 μL aliquots of supernatant were transferred for LC-MS analysis.

### HPLC conditions (T3)

All samples were collected by LC-MS system according to machine orders. The analysis conditions are as follows: UPLC: Column, Waters ACQUITY UPLC HSS T3 C18 (1.8 µ m, 2.1 mm * 100 mm); column temperature 40 °C; flow rate, 0.4 mL/min; injection volume, 2 μ L; solvent system, water (0.1% formic OPLS acid): acetonitrile (0.1% formic acid; elute the column with 5% mobile phase B (0.1% formic acid acetonitrile solution) at 0 minutes, then linearly gradient to 90% mobile phase B within 11 minutes, maintain for 1 minute, and then return to 5% mobile phase C within 0.1 minutes, maintain for 1.9 minutes.

### Analytical method

The original data file acquisited by LC-MS was converted into mzML format by ProteoWizard software. Peak extraction, peak alignment and retention time correction were respectively performed by XCMS program. The “SVR” method was used to correct the peak area. The peaks with detetion rate lower than 50% in each group of samples were discarded. After that, metabolic identification information was obtained by searching the laboratory’s self-built database, integrated public database, AI database and metDNA.

1. PCA.

Unsupervised Principal Component Analysis (PCA) is conducted using the statistical function `prcomp` in R. It is essential to perform unit variance scaling on the data prior to executing unsupervised PCA.

2. Hierarchical Cluster Analysis and Pearson Correlation Coefficients.

The results of the Hierarchical Cluster Analysis (HCA) for samples and metabolites are presented in a heatmap alongside a dendrogram. In contrast, the Pearson correlation coefficient (PCC) between samples is calculated using the cor function in R and is represented solely by the heatmap. Both HCA and PCC analyses are conducted using the R package ‘Complex Heatmap’. For HCA, the normalized signal intensity of metabolites is visualized on a unit variance scale.

3. Selected differential metabolites.

For the two analyses, differential metabolites were identified based on a VIP threshold (VIP > 1) and a P-value threshold (P < 0.05, Student’s t-test). The VIP values were derived from the OPLS-DA (Orthogonal Partial Least Squares-Discriminant Analysis) results, which also included score and permutation graphs generated using the R package MetaboAnalystR. Before conducting OPLS-DA, the data were subjected to logarithmic transformation (log2) and mean centering. To prevent overfitting, a permutation test with 200 permutations was performed.

### Statistical analysis

Statistical analysis was conducted using SPSS 26.0 software. Normal distribution and mean square deviation measurement data are represented as mean ± standard deviation. When testing intestinal tissue samples, independent sample t-test is used for comparison between the two groups. The comparison between three groups undergoing surgery at different times after radiotherapy was conducted using one-way analysis of variance. Independent sample t-test was used to compare serum inflammatory factors between patients who received radiotherapy and those who did not. The median (quartiles) is used for comparison between groups that do not meet the above criteria. * p< 0.05; **p < 0.01; △p > 0.05.

## Result

### Post-radiotherapy intestinal mucosal damage and repair pattern

**Figure 1:**
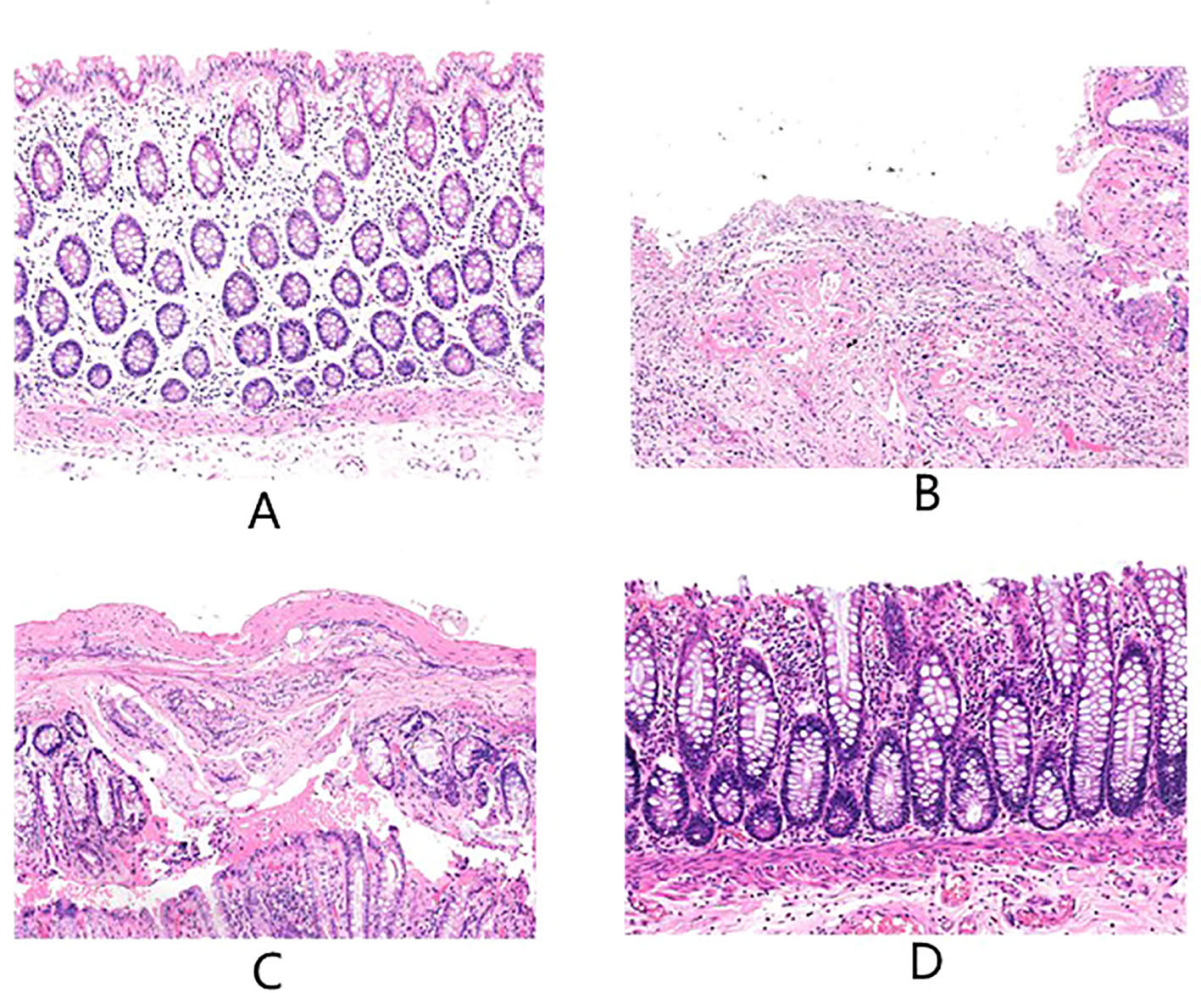
The above are the more representative pictures of HE stained slices of normal tissue intestinal mucosa distal to the tumor in the postoperative period of patients with rectal cancer who underwent surgery in our center, **(A-D)** are 60 days ± 3 days, 75 days ± 3 days, and 90 days ± 3 days after no radiotherapy versus radiotherapy, respectively.

After radiotherapy, HE staining showed obvious damage to the intestinal mucosa, intestinal mucosal destruction, inflammatory cell infiltration, and original structure changes. Moreover, with the prolongation of the time after radiotherapy, there was gradually reduced intestinal mucosal damage. But different degrees of damage persisted in about 12 weeks after radiotherapy. This pattern of change was revealed in HE-stained tissue sections. **Figures 1A–D** are the more representative HE-stained slices of the postoperative intestinal mucosa of patients from the four groups. In Figure A, it was observed with mild proctitis, with intact epithelial structure, and a small amount of chronic inflammatory cells infiltrating the mesenchymal stroma. in Figure B, there were severe active chronic proctitis, with ulcer formation and granulomatous tissue hyperplasia, as well as apoptotic crypts. Moreover, in this figure partially, there existed active chronic proctitis with ulcer formation and granulomatous tissue proliferation, crypt apoptosis in some parts of the surface epithelium with reparative changes, interstitial fibrosis, massive lymphocytes and plasma cells, capillary dilatation, as well as congestion with hemorrhage. In Figure C, there was active chronic proctitis with crypt apoptosis, proliferative interstitial fibrous tissue with fibrosis and infiltration of lymphocytes and plasma cells, swelling of endothelial cells of small submucosal arteries, and formation of occlusive vasculitis. In addition, in Figure D, there were intestinal mucosa with moderate inflammation and erosion, infiltration of inflammatory cells in the interstitium, and surface epithelial loss.

### Patterns of INF-γ and TGF-β changes in intestinal mucosa

**Figure 2:**
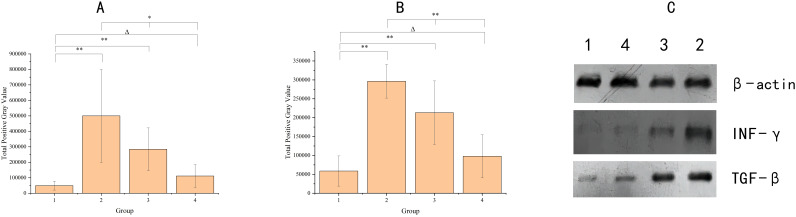
The graph shows the cytokine levels in the intestinal mucosal tissues of the four groups of samples. Panel **(A)** indicates INF-γ, Panel **(B)** indicates TGF-β, X-axis groups, where group 1 (no radiotherapy group), group 2 (60 ± 3 days after radiotherapy), group 3 (75 ± 3 days after radiotherapy), and group 4 (90 ± 3 days after radiotherapy). y-axis indicates the total grayscale values of the WB detection bands for each cytokine. Where * denotes p< 0.05; ** denotes p < 0.01; Δ denotes p > 0.05. Panel **(C)** shows the electrophoretic images of cytokines detected by protein blotting, with different cytokines indicated on the right side and different groups labeled on the top.

As shown in **Figure 2**, the levels of INF-γ and TGF-β in the postoperative intestinal mucosal tissue were significantly elevated within the 8–12 week range after radiotherapy compared to the non-radiotherapy group. Over time, INF-γ and TGF-β levels gradually returned to the normal range, and did not completely normalize within the 8–12 week period. However, there was no longer statistically significant difference in INF-γ and TGF-β levels by 90 days post-radiotherapy. Independent sample t-tests and one-way ANOVA were respectively employed for comparisons between the non-radiotherapy group and various post-radiotherapy intervals, as well as those among the different post-radiotherapy intervals.

### Post-radiotherapy changes in serum levels of IL-1β, IL-6, IL-17, and INF-γ in patients 8–12 weeks

**Figure 3:**
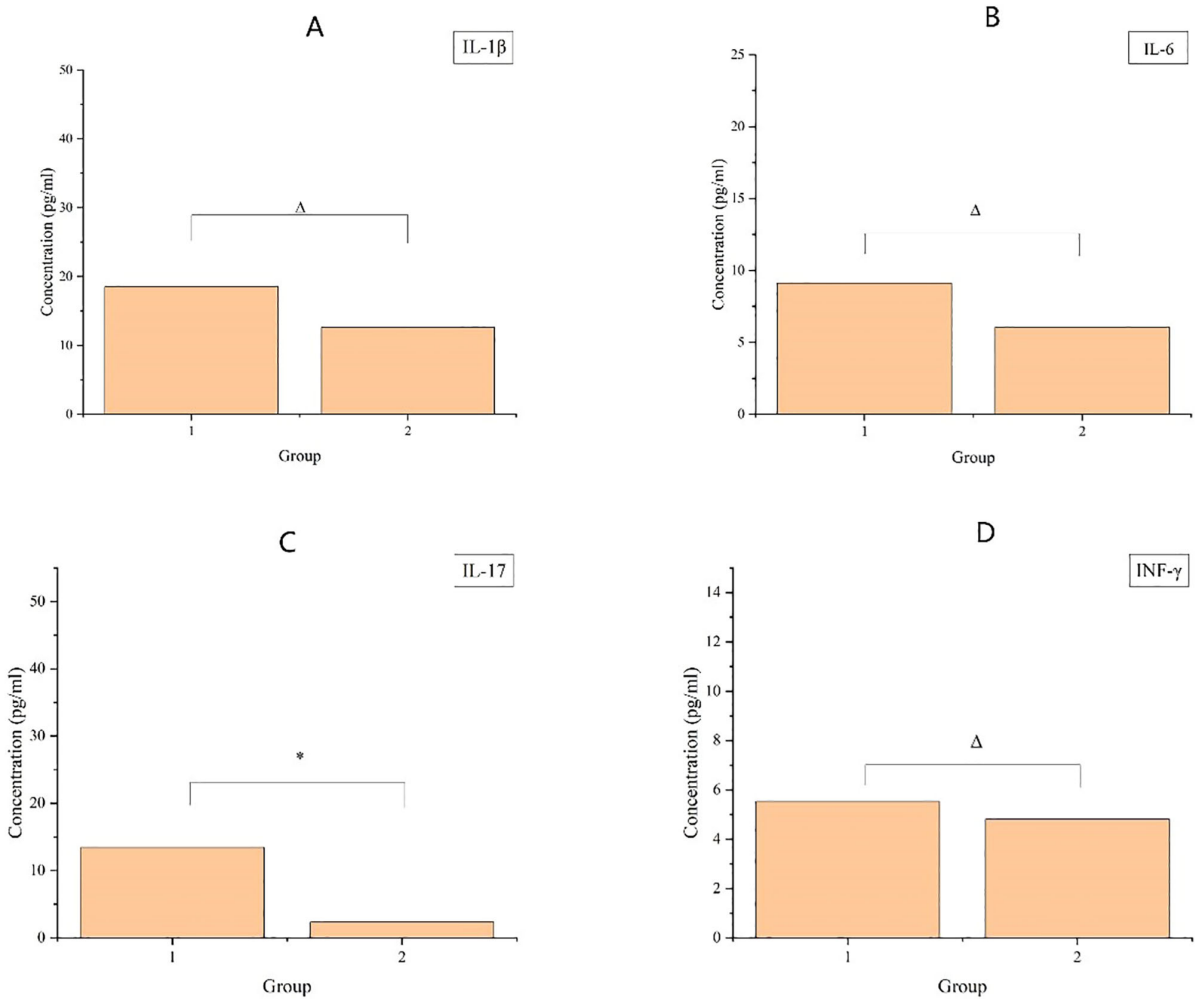
The upper graph demonstrates the cytokine content in patients’ serum, where **(A-D)** denote the four inflammatory factors, IL-1β, IL-6, IL-17, and INF-γ, respectively, with vertical coordinates denoting the content, and horizontal coordinates denoting the groups, Group 1 (8–12 weeks after radiotherapy) and Group 2 (no radiotherapy group). Where * indicates p< 0.05; ** indicates p < 0.01; Δ indicates p > 0.05.

As displayed in **Figure 3**, compared with patients who did not receive preoperative radiotherapy, patients who received preoperative radiotherapy had higher serum levels of IL-1 β, IL-6, IL-17, and INF-γ. However, no statistically significant differences were observed in the levels of IL-1 β, IL-6, and INF-γ. IL-17 levels showed statistically significant differences.

### Differential metabolites in saliva

**Figure 4:**
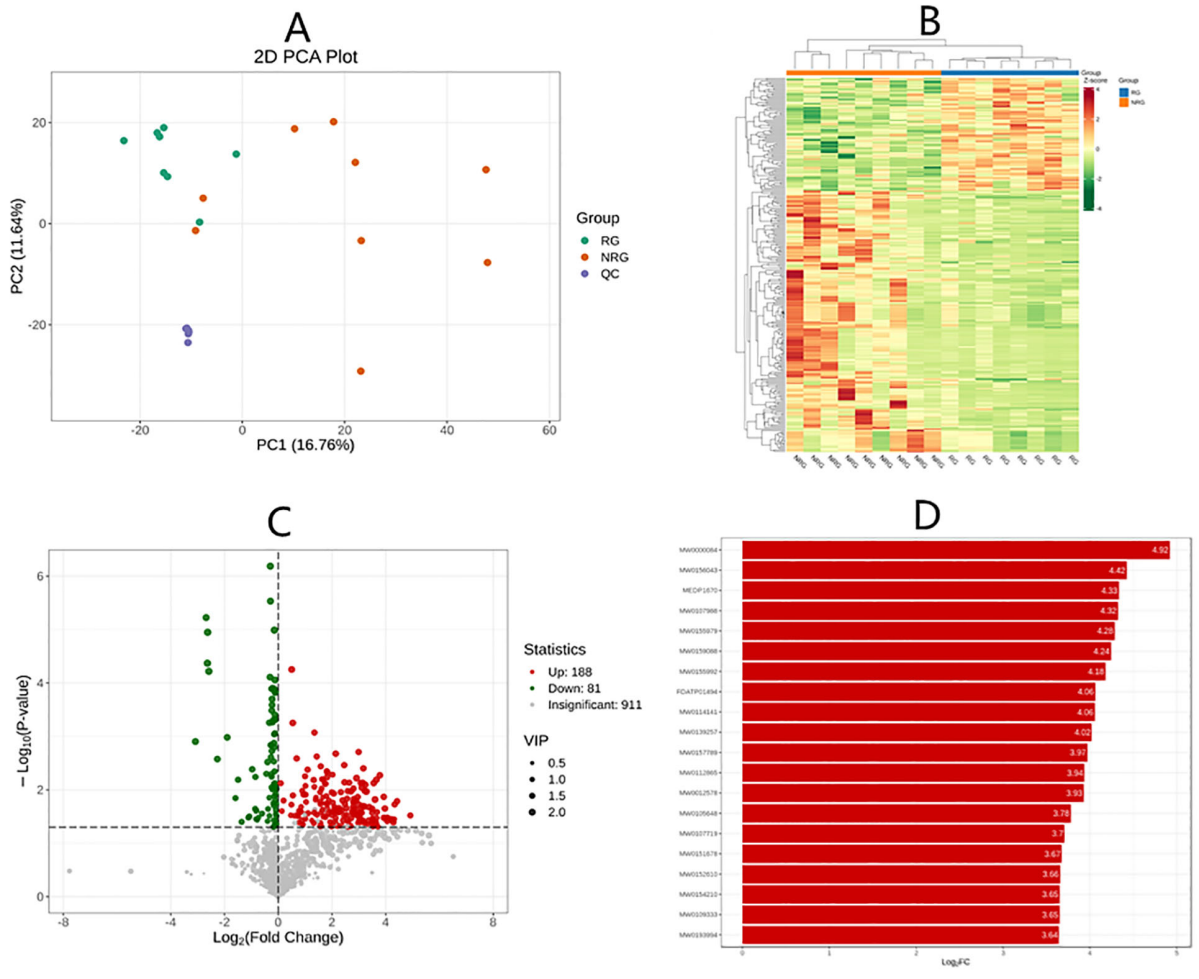
Panel **(A)** is the grouped principal component analysis, PC1 represents the first principal component, PC2 represents the second principal component, and the percentage represents the variance explained by the principal component to the data set; each point in the figure represents a sample, and samples in the same group are represented by the same color, and Group is the subgroup. Panel **(B)** is the differential metabolite clustering heatmap, the horizontal is the information of the samples, the vertical is the information of the differential metabolites, and Group is the subgroup. Group is the grouping, and different colors are the colors filled with different values obtained from the normalization of different relative contents (red for high content, green for low content). Panel **(C)** is the differential metabolite volcano plot, each point represents a metabolite, where green points represent down-regulated differential metabolites and red points represent up-regulated differential metabolites; the horizontal coordinate represents the relative content of a metabolite in the two groups of samples Under VIP + FC + P-value screening condition: the vertical coordinate represents the significance level of difference (-log1010P-value), and the size of the dots represents the VIP value. Panel **(D)** is the bar chart of differential metabolites, and the horizontal coordinate is the log22FC of the differential metabolites, i.e., the value of the multiplicity of difference of differential metabolites logarithmically based on the base of 2, and the vertical coordinate is the differential metabolites. The vertical coordinate is the differential metabolite. Red color represents up-regulation of metabolite content and green color represents down-regulation of metabolite content.

This study continued to examine the differences of metabolites in saliva by non-targeted metabolomics, and the results are shown in **Figure 4**. Specifically, **Figure 4A** is the main component analysis map, and **Figure 4B** is the clustering heat map of differential metabolites. There was significant difference of differential metabolites between the radiotherapy group (RG) and the non-radiotherapy group (NRG). **Figure 4C** is a volcano plot of differential metabolites, with red dots indicating upregulated metabolites and green dots indicating downregulated metabolites. There were more up-regulated/down-regulated differential metabolites in CRC patients after radiotherapy compared with those without radiotherapy. Figure D shows the bar chart of the multiplicity of difference, with red representing the up-regulation of metabolite content, and green indicating the down-regulation of metabolite content. This study detected the top-20 metabolites in the multiplicity of difference in the comparison between groups, all of which were up-regulated. Among them, most metabolites were amino acids and their metabolites, including 3-hydroxyquinidine, prolyl-valyl-aspartic acid, glycine-glycine-phenylalanine, Lysyl alanyl-alanine, prolyl-prolinyl-leucine, valine-lysinyl-glutamic acid, and prolyl-serinyl-isoleucine.

## Discussion

CRC remains one of the most prevalent gastrointestinal malignancies. Advances in awareness and early screening have improved patient staging of CRC, however, many patients are still diagnosed in the intermediate to advanced stages (11, 12). For individuals with stage II-III CRC, preoperative radiotherapy is widely recognized as an effective treatment. It significantly reduces the likelihood of recurrence and metastasis, and increases the success rate of anal preservation in patients with lower CRC (13). However, radiotherapy is a double-edged sword, which presents challenges, such as radiation-induced enteritis, even with anti-oncological benefits. This condition complicates pelvic radiotherapy for both patients and surgeons. Patients may experience complications such as abdominal adhesions, intestinal edema, increased fragility, and impaired healing, ultimately making surgical procedures more difficult and risky. Moreover, it compromises the intestinal mucosal barrier, potentially resulting in bacterial translocation, increased postoperative systemic inflammatory responses, and elevated risks of anastomotic fistulas. This radiation damage also restricts the feasible dose of radiotherapy, significantly decreasing the quality of life for treated cancer patients (14–16).

In patients with CRC, the first priority is usually oncological outcomes, with insufficient attention given to maintaining the integrity of the intestinal barrier. However, it cannot be ignored that radiotherapy can cause damage to the intestinal mucosa and the resultant complications. The intestinal barrier comprises mechanical, chemical, biological, and immune components.

Radiotherapy has been documented to disrupt this barrier, manifesting as intestinal edema, villous atrophy, compromised tight junctions, as well as reduced absorptive and barrier functions (17–19). Zhao et al. demonstrated an correlation of the mechanical barrier disruption with decreased levels of Claudin family proteins and tight junction protein NO-1 (20). Moreover, our previous research revealed that damage to the intestinal mucosal immune barrier function might alter the proportion of CD4+and CD8+T lymphocyte subsets; decrease the content of secretory immunoglobulin A; as well as elevate levels of pro-inflammatory cytokines IL-1β, IL-6, and IL-17 in intestinal tissue. Previous studies in patients with inflammatory bowel disease have found that strengthening the intestinal mucosal barrier function is closely related to the IL-17RA pathway (21). It can be inferred that there was a compromise in the immune barrier and an onset of radiation-induced enterocolitis. Notably, alterations in oral flora composition and abundance were found in irradiated CRC patients compared to non-irradiated individuals, suggesting a connection to the intestinal mucosal biological barrier. Given the known association between oral microbiota and systemic diseases, Jiaming Zhang et al. found correlations between altered oral and intestinal flora in thyroid cancer patients (22, 23), while Xiaoxiao Li et al. summarized numerous studies on the interplay between gastrointestinal malignancies and oral microbiota (24).

In our study, HE staining revealed that in CRC patients undergoing resection shortly after radiotherapy exhibited, the intestinal mucosa was observed with severe active chronic proctitis, characterized by ulceration, granulomatous tissue formation, crypt apoptosis, reparative changes in surface epithelium, interstitial fibrosis, as well as lymphocyte and plasma cell infiltration, alongside capillary dilation and hemorrhage. All these changes might be recovered over time, culminating in improved tissue architecture and reduced inflammatory response. However, the intestinal mucosa of these patients did not return to normal levels at least 12 weeks post-radiotherapy, suggesting the possibility of recovery, but no complete recovery potentially.

Meanwhile, increased levels of INF-γ and TGF-β were noted in the intestinal mucosa, with time-dependent amelioration. INF-γ serves as a significant immunomodulatory protein with diverse functions, including promotion of intestinal barrier damage, with elevated levels under conditions such as infections, inflammation, tumors, etc. (25). TGF-β, a multifunctional cytokine from the TGF-β superfamily, regulates several cellular processes and is integral to the repair of intestinal barrier damage and colitis (26). Therefore, in addition to presence of radiation enteritis, increased INF-γ and TGF-β levels in post-radiotherapy patients may reveal dysfunction of the intestinal mucosal immune barrier, which gradually repairs over time.

We also studied the levels of inflammatory factors (i.e., IL-1β, IL-6, IL-17, and INF-γ) in the serum of CRC patients. All the four cytokines were significantly elevated, especially IL-17. IL-17 is a cytokine produced by activated CD4+T cells, which participates in the body’s immune defense and inflammatory response (27). This may reinforce our previous findings regarding increased CD4+ T lymphocyte proportions and correlating IL-17 levels in intestinal tissues post-radiotherapy, likely tied to declined barrier function and release of inflammatory mediators. Monitoring intestinal inflammation and repair of barrier damage via serum markers could pave the way for a succinct understanding of inflammatory responses and the recovery of intestinal mucosal integrity.

Furthermore and noticeably, radiotherapy can inflict substantial damage on the intestinal mucosal barrier, significantly affecting mechanical and immune functions, which, while partially reversible over time, may not normalize within the commonly recommended surgical time-frame of 8–12 weeks post-radiotherapy. Therefore, extending the waiting period for surgery could potentially mitigate the risk of intestinal barrier damage, contingent upon patients’ overall conditions. In addition, elevated IL-17 serum levels could also serve as a predictor to assess the recovery status of the intestinal mucosal barrier prior to surgery, helping to determining the optimal surgical timing.

Non-targeted metabolomics analyses unveiled shifts in salivary metabolites among patients post-radiotherapy, with the majority of upregulated metabolites falling within the category of amino acids and their derivatives. These metabolites have been found to exert roles of intestinal barrier repair, inflammation, and gastrointestinal tumor development (28, 29). For instance, butyric acid can enhance tight junction integrity in intestinal epithelial cells, maintaining mucosal barrier function—a process impaired in inflammatory bowel disease due to reductions in butyric acid-producing bacteria (30–32).

Changes in oral microbiota and their metabolites exhibit potential association with various systemic diseases and tumors (33–35). Dong et al. discovered alterations in the oral microbiota of mice with colon cancer after radiotherapy, which might affect tumor development and prognosis (36). Previous studies have found that chronic inflammation of the intestine may be associated with the occurrence of tumors (37). Our previous research also noted changes in the oral microbiota of patients who underwent surgery after radiotherapy and those who underwent surgery directly without radiotherapy, which were associated with upregulation of various intestinal metabolites (10). Thus, intestinal mucosal barrier damage may have a relationship with altered salivary metabolites, offering potential insights into predicting patient responses to radiotherapy and oncological outcomes.

In view of the above discussion, damage to the intestinal barrier can manifest through mechanical, biological, and immune mechanisms, and can be assessed via biopsy pathology alongside metabolite and inflammatory marker analyses. Intestinal barrier function may be assessed in a non-invasive manner of monitoring changes in oral microbiota metabolites and inflammatory factors in the bloodstream. This exploratory study may lay groundwork for future non-invasive monitoring strategies, despite the deficiency of limited sample size that imposed challenges in accurately identifying indices and ranges. Advancements in artificial intelligence and large-sample analyses may further facilitate the determination of comprehensive standards for monitoring specific saliva substance levels alongside routine biochemical markers. It can eventually benefit dynamic management of patients undergoing various radiotherapy regimens, ultimately reducing complications associated with intestinal barrier damage and enhancing surgical outcomes.

## Conclusion

Radiotherapy can cause serious damage to the intestinal mucosa and its barrier function, as well as to the immune and biological barrier functions, as evidenced by changes in histopathology, intestinal mucosa and saliva metabolites. It is possible to noninvasively detect the recovery of intestinal mucosal barrier function after radiotherapy-induced injury.

## Data Availability

The original contributions presented in the study are included in the article/**Supplementary Material**. Further inquiries can be directed to the corresponding authors.
