# Supplementary figures and images for: Radiation therapy induced intestinal barrier damage and repair process - differences in salivary metabolites and monitoring of intestinal barrier function
Source: Front Immunol. 2025 Jun 12;16:1590219. doi: 10.3389/fimmu.2025.1590219 (PMC12197936; doi:10.3389/fimmu.2025.1590219)

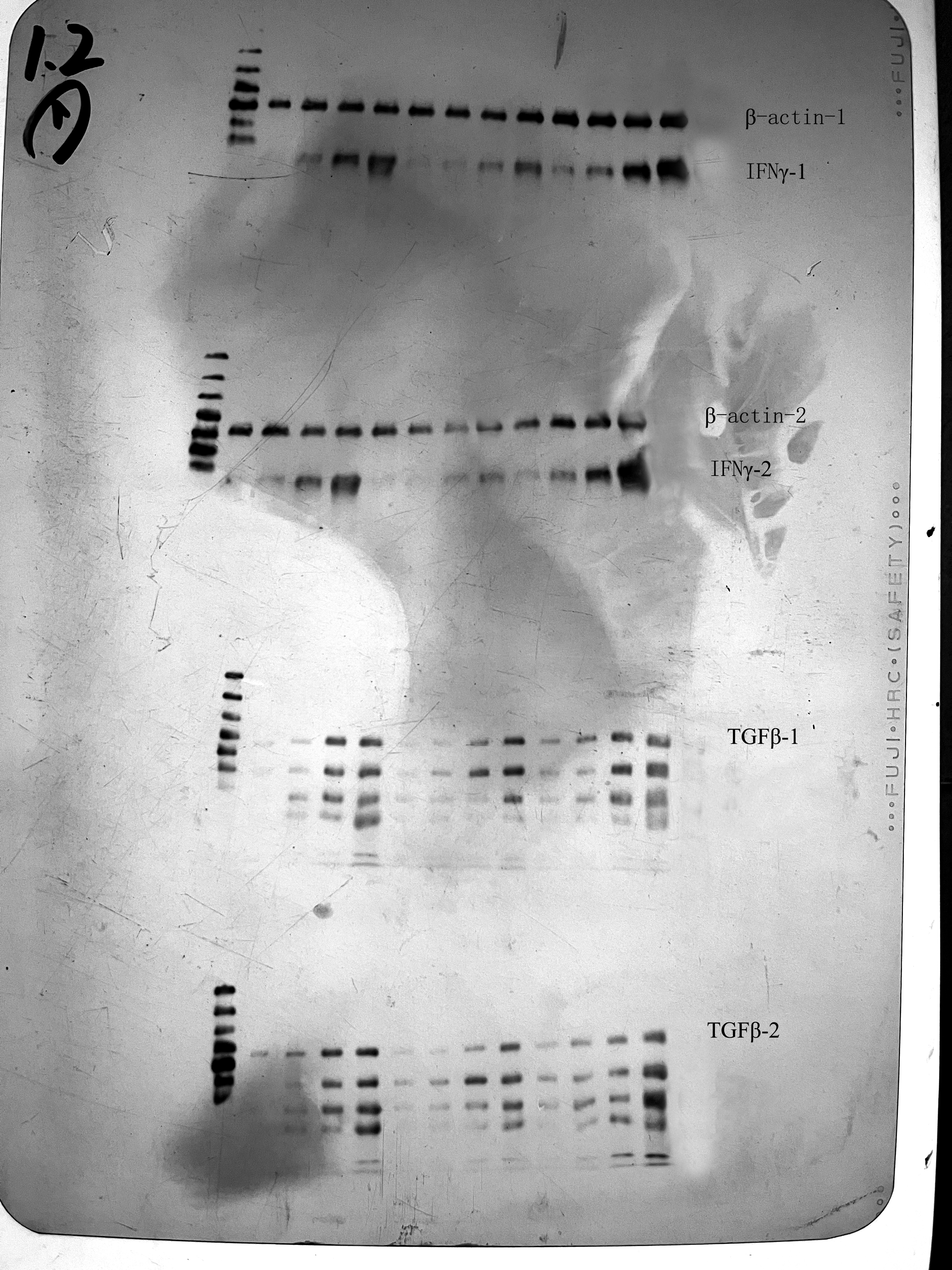

Supplement: Supplementary file 1 [file Image1.jpeg]
