# Supplementary material for: Radiation therapy induced intestinal barrier damage and repair process - differences in salivary metabolites and monitoring of intestinal barrier function
Source: Front Immunol. 2025 Jun 12;16:1590219. doi: 10.3389/fimmu.2025.1590219 (PMC12197936; doi:10.3389/fimmu.2025.1590219)
Supplement: Supplementary file 2 [file Table1.docx]

Table1-1(Saliva metabolite detection)

| Characteristic | RG(n=11) | NRG(n=13) | P |
| --- | --- | --- | --- |
| [Sex](file:///D:\%E6%96%B0%E5%BB%BA%E6%96%87%E4%BB%B6%E5%A4%B9%20(3)\%E7%99%BE%E5%BA%A6%E7%BF%BB%E8%AF%91\baidu-translate-client\resources\app.asar\app.html) | 11(54.5%) | 13(53.8%) | NS |
| Age | 63.45±10.68 | 60.85±7.68 | NS |
| BMI | 24.60±2.22 | 24.11±3.28 | NS |
| CEA | 8.03±19.64 | 2.46±1.76 | NS |
| CA-199 | 14.02±24.38 | 12.44±7.58 | NS |
| [TG](file:///D:\%E6%96%B0%E5%BB%BA%E6%96%87%E4%BB%B6%E5%A4%B9%20(3)\%E7%99%BE%E5%BA%A6%E7%BF%BB%E8%AF%91\baidu-translate-client\resources\app.asar\app.html) | 1.62±1.38 | 1.15±0.50 | NS |
| HDL | 1.32±0.29 | 1.46±0.41 | NS |
| LDL | 3.34±0.96 | 2.75±0.41 | NS |

Table1-2(Inflammatory factors of intestinal mucosa inflammation)

| Characteristic | Group1(n=6) | Group2(n=6) | Group3(n=6) | Group4(n=6) | P |
| --- | --- | --- | --- | --- | --- |
| [Sex](file:///D:\%E6%96%B0%E5%BB%BA%E6%96%87%E4%BB%B6%E5%A4%B9%20(3)\%E7%99%BE%E5%BA%A6%E7%BF%BB%E8%AF%91\baidu-translate-client\resources\app.asar\app.html) | 6(50%) | 6(50%) | 6(50%) | 6(50%) | NS |
| Age | 63.67±8.71 | 63.33±8.33 | 65.8±11.41 | 63.50±8.02 | NS |
| BMI | 27.57±1.88 | 27.18±2.89 | 27.5±0.88 | 26.78±2.02 | NS |
| Radiotherapy | 5x5 Gy | 5x5 Gy | 5x5 Gy | 5x5 Gy | NS |

Table1-3(Serum inflammatory factors)

| Characteristic | RG(n=23) | NRG(n=57) | P |
| --- | --- | --- | --- |
| [Sex](file:///D:\%E6%96%B0%E5%BB%BA%E6%96%87%E4%BB%B6%E5%A4%B9%20(3)\%E7%99%BE%E5%BA%A6%E7%BF%BB%E8%AF%91\baidu-translate-client\resources\app.asar\app.html) | 23（56.5%） | 57（57.9%） | NS |
| Age | 62.09±8.00 | 66.74±11.00 | NS |
| BMI | 26.9±1.99 | 27.0±2.50 | NS |
